# One-year outcomes of drug-coated balloon treatment for long femoropopliteal lesions: a multicentre cohort and real-world study

**DOI:** 10.1186/s12872-021-02127-x

**Published:** 2021-07-03

**Authors:** Xiaoxi Yu, Xin Zhang, Zhichao Lai, Jiang Shao, Rong Zeng, Wei Ye, Yuexin Chen, Bihui Zhang, Bo Ma, Wenteng Cao, Xiaolong Liu, Jinghui Yuan, Yuehong Zheng, Min Yang, Zhidong Ye, Bao Liu

**Affiliations:** 1 Department of Vascular Surgery, Peking Union Medical College Hospital, Peking Union Medical College and Chinese Academy of Medical Sciences, ShuaiFuYuan 1st, DongCheng-Qu, Beijing, 100730 China; 2 Department of Interventional Radiology and Vascular Surgery, Peking University First Hospital, Xishiku Street 8th, XiCheng-Qu, Beijing, 100034 China; 3 Department of Cardiovascular Surgery, China-Japan Friendship Hospital, Yinghuayuan East Street 2nd, ChaoYang-Qu, Beijing, 100029 China

**Keywords:** Peripheral artery disease, Drug-coated balloon, Femoropopliteal lesions, Real-world study, Long lesions

## Abstract

**Background:**

Drug-coated balloons (DCBs) have shown superiority in the endovascular treatment of short femoropopliteal artery disease. Few studies have focused on outcomes in long lesions. This study aimed to evaluate the safety and effectiveness of Orchid^®^ DCBs in long lesions over 1 year of follow-up.

**Methods:**

This study is a multicentre cohort and real-world study. The patients had lesions longer than or equal to 150 mm of the femoropopliteal artery and were revascularized with DCBs. The primary endpoints were primary patency, freedom from clinically driven target lesion revascularization (TLR) at 12 months and major adverse events (all-cause death and major target limb amputation). The secondary endpoints were the changes in Rutherford classification and the ankle brachial index (ABI).

**Results:**

One hundred fifteen lesions in 109 patients (mean age 67 ± 11 years, male proportion 71.6%) were included in this study. The mean lesion length was 252.3 ± 55.4 mm, and 78.3% of the lesions were chronic total occlusion (CTO). Primary patency by Kaplan–Meier estimation was 98.1% at 6 months and 82.1% at 12 months. The rate of freedom from TLR by Kaplan–Meier estimation was 88.4% through 12 months. There were no procedure- or device-related deaths through 12 months. The rate of all-cause death was 2.8%. Cox regression analysis suggested that renal failure and critical limb ischaemia (CLI) were statistically significant predictors of the primary patency endpoint.

**Conclusion:**

In our real-world study, DCBs were safe and effective when used in long femoropopliteal lesions, and the primary patency rate at 12 months by Kaplan–Meier estimation was 82.1%.

## Introduction

Numerous studies have shown satisfactory outcomes using drug-coated balloons (DCBs) in the endovascular treatment of short femoropopliteal artery disease [1–6]. These outcomes cannot be directly extrapolated to long lesions. The treatment of long lesions is known to be challenging, since long lesions are always associated with complex and calcified plaques leading to insufficient dilation and elastic recoil [7] Long lesions of the femoropopliteal artery are very common in clinical practice, and the optimal treatment remains to be explored. Furthermore, longer balloons contain more paclitaxel, and some studies suggest this may trigger dose-related adverse events, while others found different results [8–11] Therefore, the effectiveness and safety of DCBs in long lesions needs further evaluation.

The advantages of DCBs in the treatment of long lesions include inhibition of vascular intima hyperplasia without leaving anything behind, thus reducing restenosis rates and avoiding implant rupture [12, 13]. However, few clinical results have focused on long lesions in femoropopliteal peripheral artery disease, and even fewer real-world studies have been conducted [14–17]. The SFA-Long study reported a patency rate of 89.3% by Kaplan–Meier estimate at 12 months, while the primary patency in the IN.PACT Global Study Long Lesion Imaging Cohort was 91.1% [14, 16]. Most of these trials were industry-sponsored, in which patients may be highly selected and the intervention process may be tightly controlled, suggesting an ideal effect. Real-world studies with relatively loose inclusion criteria reflect real clinical situations and can reflect the actual effectiveness of the intervention. For example, in actual clinical applications, there are various methods of vessel preparation for different types of blood vessels, and studies that strictly control vessel preparation cannot reflect the actual effectiveness [18].

This study was designed in our 3 major medical centres to estimate the effectiveness and safety of Orchid^®^ DCB in long lesions (≥150 mm) through a 1-year follow-up under real-world conditions.

## Methods

This study is a multicentre, real-world, cohort study. This study analysed the clinical outcomes of patients with long femoropopliteal lesions treated with Orchid^®^ DCB (Acotec Scientific, Beijing, China) angioplasty from July 2016 to June 2019. The data were collected at 3 medical centres. The local Institutional Review Board approved this study and written informed consent was obtained from all of the participants. All methods were carried out in accordance with the relevant guidelines and regulations.

## Patients

Adult patients undergoing DCB treatment for stenotic arteriosclerotic lesions in the superficial femoral artery and/or popliteal artery were included, with stenotic or occlusive lesions for a total length of ≥ 150 mm. Multiple adjacent lesions with interval angiographically healthy segments shorter than 3 cm were considered and treated as a single lesion. The Rutherford stages ranged from 2 to 5 (claudication, resting pain or small ulcers). All included patients had inflow vessels with < 50% diameter stenosis before the treatment of femoropopliteal lesions. Patients who were allergic to paclitaxel, whose life expectancy was less than 2 years, or who had vascular surgery 6 weeks before this treatment were excluded. If there were other nonatherosclerotic vascular diseases in the target lesion, including aneurysms or vasculitis, the patients were also excluded.

### Procedures and devices

All patients had a thorough clinical examination at baseline. Each patient took 100 mg/day aspirin and 75 mg/day clopidogrel for at least 7 days before surgery. If the patients had no history of aspirin or clopidogrel, they took 300 mg within 12 hours before surgery.

During the procedure, 75 IU/kg heparin was administered after sheath insertion. Thrombectomy was performed in some patients before predilation if thrombosis was suspected based on the clinical symptoms and radiographic evidence. Predilation (2 minutes) was routinely performed with uncoated balloons 0.5 to 1 mm smaller than the reference vessel. The intraluminal or subintimal approach was at the discretion of the operators. Atherectomy was performed only under necessary conditions determined by the operator; for example, the calcification was so severe or near the joint that predilation was not satisfactory. The DCBs were Orchid^®^ from the Acotec Scientific Company, ranging from 4-6 mm in diameter and 120-300 mm in length, and the dose of paclitaxel in Orchid^®^ DCBs was 3.0 µg/mm^2^.[19] The DCBs were 1.0 mm larger than the uncoated balloon in diameter and were inflated only once for 3 minutes at 6-12 atmospheres. There were lesions of approximately 400 mm, but the longest balloon accessible was 300 mm. Therefore, if two or more DCBs were needed for only one lesion, they must overlap by more than 5 mm. If there was any residual stenosis (> 30%) or flow limiting dissection, another expansion of the uncoated balloon was performed. After repeated dilation, a bailout stent was deployed during the same intervention if persistent stenosis or flow-limiting dissection still existed. The bailout stents used included Zilver Flex^®^ (Cook, Bjaeverskov, Denmark), Protégé Everflex^TM^ (Covidien, Plymouth, Minn), LifeStent^®^ (Bard, Tempe, AZ), and Innova^TM^ (Boston Scientific, Marlborough, MA). Inflow and outflow lesions were also treated during the same intervention at the discretion of the operators.

After revascularization, patients were prescribed 100 mg/day aspirin and 75 mg/day clopidogrel for at least 12 weeks. Follow-up information was obtained by telephone and the outpatient service at 3 months, 6 months, and 1 year after DCB angioplasty, including the symptoms, Rutherford classification, adverse events, ankle brachial index (ABI), and imaging results. Computed tomography angiography (CTA) and Doppler ultrasound examination (US) were recommended at 6 and 12 months and in the event of ischaemic symptoms.

### Definition and study endpoints

Chronic total occlusion (CTO) was defined as the minimum lumen diameter of the target lesion being 0. Calcification of the lesions was evaluated using the PACSS scale [20]. A moderate to severe degree was defined as calcification on two sides of the vascular walls, and one of them was longer than 5 cm. Critical limb ischaemia (CLI) was defined as the presence of ischaemic rest pain, nonhealing wounds/ulcers, or gangrene for >2 weeks with associated evidence of hypoperfusion according to the American Heart Association (AHA) guidelines [21]. Device success was defined as successful access of the guidewire and accurate deployment of the balloons to the target lesion, while technical success was defined as both device success and residual stenosis < 30% after the endovascular procedure.

The primary endpoints in this study were primary patency, freedom from clinically driven target lesion revascularization (TLR) and major adverse events (all-cause death and major target limb amputation). Primary patency is defined as freedom from restenosis (> 50% residual lumen diameters in the target lesion under CTA, or peak systolic velocity ratio ≤ 2.4 under Doppler ultrasound examination) and freedom from clinically driven TLR. Clinically driven TLR is defined as any revascularization of the target lesion because of apparent symptoms or the imaging results mentioned above. The secondary endpoints were the change in Rutherford classification and ABI between preprocedure and follow-up.

### Statistical analysis

The data were analysed on the per-protocol population. Descriptive statistics were used to estimate values and changes from baseline as the absolute frequency (percentage) for categorical variables and mean ± SD for continuous variables.

Primary patency and freedom from TLR were estimated by Kaplan–Meier analyses. Comparison of the Rutherford classification and ABI between the preprocedure and follow-up was tested through the Wilcoxon signed rank-sum test. In the univariate subgroup analysis of primary patency, the log-rank test of Kaplan–Meier survival curves was used. Variables with a *p* value < 0.1 were included in the multivariate analysis. Cox proportional hazards multivariate regression analysis was used to estimate the independent influence of the potential prognostic factors. Statistical significance is defined as *p* values < 0.05. Data were analysed with SPSS version 25.0 (SPSS, Chicago, Illinois) for Windows.

## Results

### Characteristics and procedural details

This study included 115 lesions in 109 patients whose mean age was 67 ± 11 years old, and the male proportion was 71.6%. Baseline clinical characteristics and lesion characteristics are shown in Table 1. The mean lesion length was 252.3 ± 55.4 mm, and 78.3% of the lesions were CTOs. In addition, 24.3% of the lesions were in-stent restenosis, and non-stent restenosis accounted for 9.6%, while the others were de novo stenoses. Runoff information was available at 2 centres (86 out of 115 lesions), and 87.2% of patients had at least one below-the-knee flow vessel. In the procedures, 1.5 ± 0.6 DCBs were used per lesion, while the bailout stent rate was 27.0%. Atherectomy/thrombectomy was used when necessary and accounted for 13.9% of the lesions (16 out of 115 lesions). All procedures were performed smoothly, with a device success rate of 100% and a technical success rate of 100%.

**Table 1:** Baseline clinical characteristics of patients and lesions

Characteristics (per subject)	(N = 109)	Characteristics (per Lesion)	
Age, years	67±11	Lesions	115
Male	71.6 (78)	Lesion type	
Hypertension	71.6 (78)	De novo	66.1 (76/115)
Hyperlipidemia	34.9 (38)	ISR^a^	24.3 (28/115)
Diabetes	59.6 (65)	Restenosis (non-stent)	9.6 (11/115)
Prior/Current smoking	46.8 (51)	Lesion length, mm	252.3 ± 55.4
Renal failure	7.3 (8)	With moderate or severe calcification	47.0 (54/115)
Coronary arterial disease	25.7 (28)	Stenosis degree	
History of stroke	18.3 (20)	Slight or moderate	0 (0/115)
Rutherford class		Severe	21.7 (25/115)
2	2.8 (3)	Total occlusion	78.3 (90/115)
3	56.0 (61)	TASC^b^ lesion type	
4	26.6 (29)	B	2.6 (3/115)
5	14.7 (16)	C	68.7 (79/115)
		D	28.7 (33/115)
		BTK^c^ flow	
		3 vessels	41.9 (36/86)
		2 vessels	20.9 (18/86)
		1 vessel	24.4 (21/86)
		None	12.8 (11/86)

Values are mean ± stand deviation (SD) or % (n) or % (n/N)

^a^ISR in-stent restenosis

^b^TASC TransAtlantic InterSociety Consensus II

^c^BTK below-the-knee, BTK data are available at 2 centers

All enrolled patients underwent at least one follow-up visit, and the mean follow-up of patients in this study was 391 ± 182 days after surgery. Eighty of 115 patients complied with the surveillance imaging, and the remaining patients completed all follow-up examinations except the imaging examinations because they thought their symptoms had completely resolved. Through detailed questioning and evaluation, every effort was made to reduce errors in the final outcome data among these patients.

### Outcomes

**Fig. 1:**
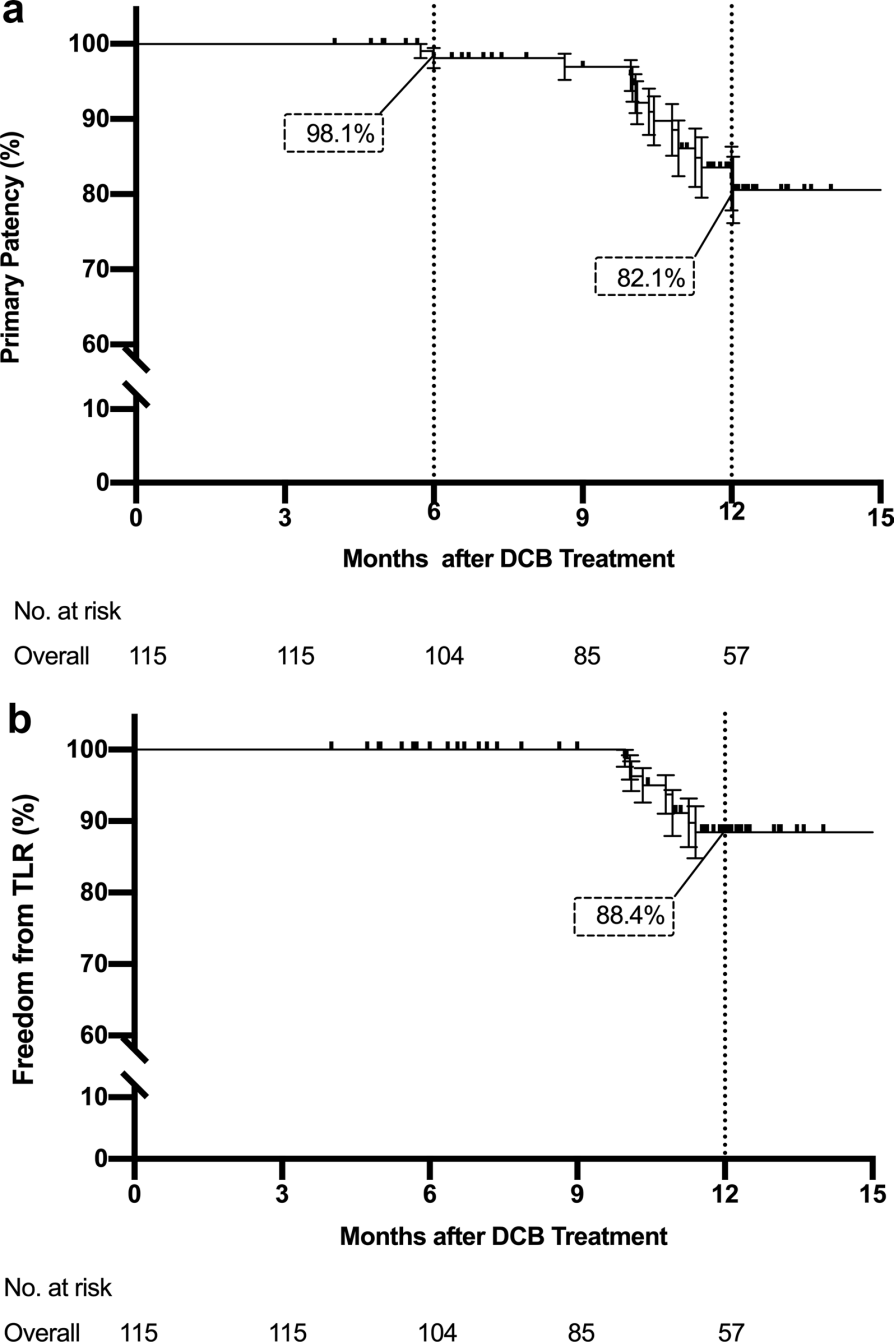
Kaplan–Meier curves of **a** Primary Patency and **b** Target Lesion Revascularization (TLR) Endpoints. Primary patency is defined as freedom from restenosis (> 50% residual lumen diameters in the target lesion under computed tomography angiography, or peak systolic velocity ratio ≤ 2.4 under Doppler ultrasound examination) and freedom from clinically driven TLR

Primary patency by Kaplan–Meier estimation was 98.1% at 6 months, while at 12 months, the patency rate was 82.1%. The rate of freedom from TLR by Kaplan–Meier estimation was 88.4% at 12 months (Fig. 1).

**Fig. 2:**
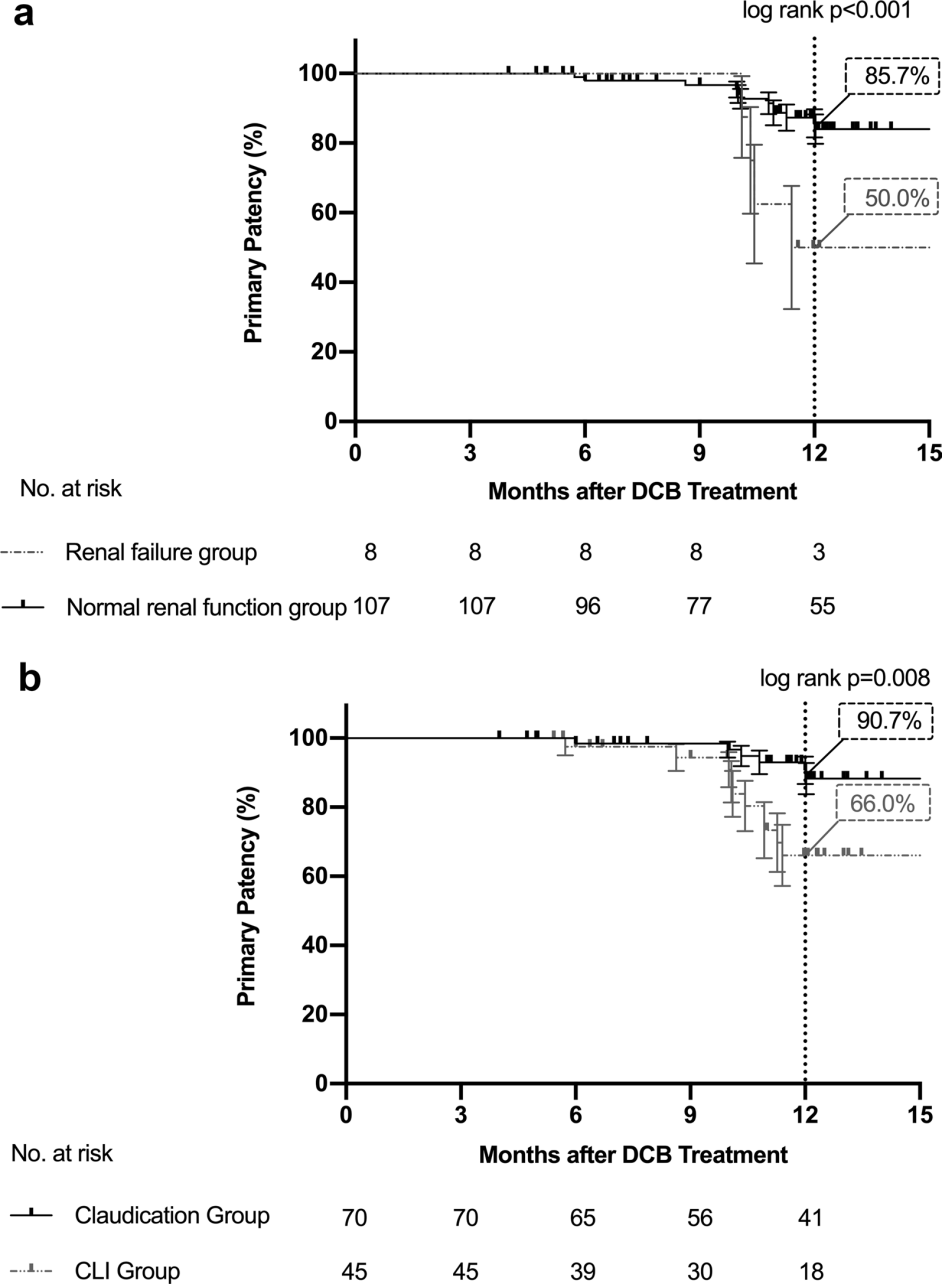
Kaplan–Meier curves of primary patency endpoints in subgroup analysis. Kaplan–Meier curves show the incidence of the primary patency endpoint over the 1-year follow-up in patients with renal failure versus patients with normal renal function (**a**) and in claudication patients versus CLI patients (**b**). The differences were statistically significant

To identify independent predictors of restenosis, subgroup comparisons were applied according to the clinical and lesion characteristics (Table 2). Primary patency by Kaplan–Meier estimation showed a significant difference between subgroups categorized by renal failure as well as by CLI (Fig. 2). The primary patency rate was only 50.0% at 12 months for patients with renal failure, while the rate was 85.7% for patients without renal failure. For CLI patients, the primary patency rate at 12 months was only 66.0%, but the rate could be as high as 90.7% for patients presenting with claudication. No significant difference was found between the other subgroups. Considering the results of the subgroup analysis and confounders, renal failure, CLI, and restenosis (in-stent and non-stent), TASC D lesions were considered variables, and a Cox proportional hazards regression model was used to analyse potential predictors (Table 3). Cox analysis suggested that renal failure and CLI were statistically significant predictors of the primary patency endpoint.

**Table 2:** Comparison of primary patency between subgroups according to the clinical and lesion characteristics

Subgroup	N	Primary patency (%)	*p* value	Subgroup	N	Primary patency (%)	*p* value
Female	31	77.0 ± 9.0	0.217	Claudication	70	90.7 ± 4.0	0.008*
Male	84	83.9 ± 4.7		CLI^a^	45	66.0 ± 8.8	
Hypertension	83	80.7 ± 5.3	0.727	Restenosis	39	73.0 ± 7.8	0.099
No Hypertension	32	80.6 ± 7.8		De novo	76	88.3 ± 4.5	
Hyperlipidemia	40	78.8 ± 7.9	0.998	ISR^b^	28	71.2 ± 9.2	0.238
No Hyperlipidemia	75	81.5 ± 5.3		Not ISR	87	86.5 ± 4.5	
Diabetes	69	80.4 ± 5.6	0.560	Moderate or severe calcification	54	87.5 ± 5.3	0.105
No Diabetes	46	84.6 ± 6.4		No or mild calcification	56	81.4 ± 6.4	
Prior/Current Smoking	52	81.2 ± 6.5	0.720	Total occlusion	90	78.0 ± 5.1	0.176
Nonsmoker	63	82.7 ± 5.6		Stenosis	25	100.0	
Coronary arterial disease	30	78.9 ± 8.4	0.763	Length > 200mm	84	77.6 ± 5.8	0.123
No coronary arterial disease	85	83.3 ± 4.9		Length ≤ 200mm	31	90.0 ± 5.5	
History of stroke	20	62.7 ± 12.1	0.173	Bailout stent	31	91.7 ± 5.6	0.291
No stroke	95	86.6 ± 4.2		No bailout stent	84	78.3 ± 5.4	
Renal failure	8	50.0 ± 17.7	0.001*	TASC^c^ D lesions	33	67.5 ± 9.6	0.066
No renal failure	107	85.7 ± 4.0		TASC B/C lesions	82	88.0 ± 4.3	
With outflow	75	92.8 ± 3.6	0.113				
No outflow	11	83.3 ± 15.2					

Values are Kaplan–Meier estimates ± SE. Log-rank test

^a^CLI critical limb ischemia

^b^ISR in-stent restenosis

^c^TASC TransAtlantic InterSociety Consensus II

^*^Statistically significant

**Table 3:** Cox regression analysis of variables affecting the primary patency endpoint

Variables	HR (95% CI)	*p* value
Renal failure	4.661 (1.550–14.016)	0.006*
CLI^a^	2.985 (1.248–7.142)	0.014*
Restenosis	1.859 (0.793–4.353)	0.154
TASC^b^ D	1.684 (0.706–4.016)	0.240

*HR* hazard ratio, *CI* confidence interval

^a^CLI critical limb ischemia

^b^TASC TransAtlantic InterSociety Consensus II

^*^Statistically significant

**Fig. 3:**
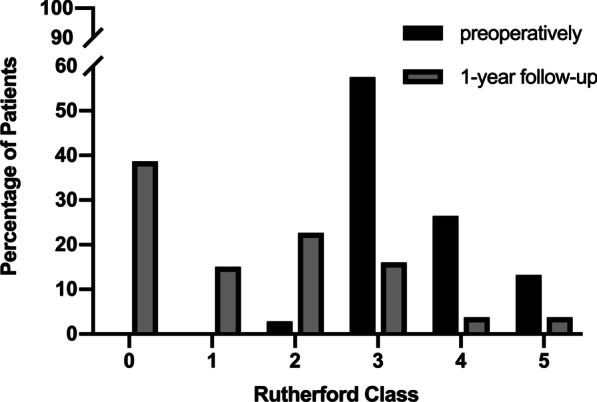
Improvement of the Rutherford classification. Rutherford's stages of enrolled patients ranged from 2 to 5 (claudication, resting pain or small ulcers) preoperatively. The changes in the Rutherford classification between baseline and 12 months were statistically significant (*p* < 0.001). The percentage of patients who had functional improvement in the Rutherford classification at 12 months was 85.8% (n = 91 of 106)

The changes in Rutherford classification between baseline and 12 months were available in 106 patients (3 patients died at the 12-month follow-up), showing a significant difference (*p* < 0.001) (Fig. 3). Rutherford classification was reduced in 85% of patients (n = 91 of 106) at 12 months. The proportion of Rutherford class 0 to 1 patients increased from 0% at baseline to 53.8% (n = 57 of 106) at 12 months, and the proportion of Rutherford class 4 to 5 patients decreased from 39.6% (n = 42 of 106) at baseline to 7.5% (n = 8 of 106) at 12 months. The mean ABI changed from 0.479 ± 0.267 before the procedures to 0.909 ± 0.479 at the 12-month follow-up, which was statistically significant (*p* < 0.001, N = 74).

Regarding safety outcomes, there were no procedure- or device-related deaths through 12 months. Major adverse events included a major amputation that occurred in 1 patient half a month after DCB treatment. The rate of all-cause death was 2.8% (n = 3 of 109) through 12 months. The causes of death in the 3 patients were myocardial infarction and heart failure.

## Discussion

As the effectiveness of DCBs in short femoropopliteal lesions has been sufficiently proven, DCBs are extensively used in short lesions. However, long lesions with femoropopliteal artery stenosis are very common, and the treatment is challenging. However, long-lesion studies started later, and the application of DCBs in long lesions is still limited. A study summarized the use of DCBs and drug-eluted stents (DESs) in 91,864 femoropopliteal lesions, finding that DCBs are mostly used in medium length, minimally calcified lesions, while DESs are mostly used in longer, more heavily calcified lesions [22] Thus, the evidence for the use of DCBs in long lesions is insufficient, and their effectiveness and safety are unclear. To address this problem, the lengths of the lesions included in this study were all longer than or equal to 150 mm, which is the boundary of TASC B and C. Meanwhile, the proportion of CTOs in our study was 78.3%, which is higher than that of other long-lesion studies (for example, 49.5% in the SFA-Long study and 60.4% in the IN.PACT Global Study Long Lesion Imaging Cohort) [14, 16]; therefore, our results better reflect the efficacy of DCBs on long and more complex lesions in the real world. Another feature of this study is that all patients were Asian, while most published studies were performed in Western countries. Potential differences between races in the composition and calcium of atherosclerotic plaques may affect the patency rate and clinical decisions [23].

According to other Orchid^®^ DCB studies applied in femoropopliteal lesions, the primary patency rate of the DCB group (n = 100, bailout stent 19.0%) in the AcoArt I trial was 76.1% at 12 months and 64.6% at 24 months, significantly improving the clinical outcomes compared with the uncoated balloon group (*p* < 0.001) [24, 25]. The mean lesion length in the DCB group in the AcoArt I trial was 147 ± 110 mm, while the mean length in this study was 252.3 ± 55.4 mm. The 1-year primary patency rate in this study was 82.1%, suggesting that Orchid^®^ DCBs are effective in treating long lesions ≥ 150 mm at 1 year. In comparison to our results, other studies focusing on long lesions, such as the SFA-Long study, reported that the primary patency was 89.3% at 12 months by Kaplan–Meier estimations (n = 105, bailout stent 10.5%) [14], while the 12-month primary patency rate of the IN.PACT Global Study Long Lesion Imaging Cohort was 91.1% (n = 164, bailout stent 39.4%) [16]. There are some possible reasons why the primary patency rate in this study is slightly lower than that in the SFA-Long study and the IN.PACT Global Study Long Lesion Imaging Cohort, including different characteristics of the patients and lesions. For example, in our study, patients with Rutherford grade 4–5 accounted for 41.3%, and restenosis (in-stent and non-stented) accounted for 33.9%, higher than the other long-lesion studies. Therefore, more clinical evidence is needed comparing the effectiveness of different types of DCBs.

Among patients categorized by different clinical and lesion characteristics, subgroup analysis was performed to study the potential risk factors related to primary patency. These results suggested that subgroups categorized by renal failure and CLI showed significant differences in the patency rate at 12 months. In the Cox regression analysis, we found that these 2 variables were also independent predictors, suggesting that patients with renal failure or CLI had worse outcomes. CLI is known to be an end-stage form of peripheral artery disease with poor clinical outcomes, and thus, it is reasonable that the patency rate in the CLI group was lower. In regard to the negative effect of renal failure, many studies have confirmed the acceleration of atherosclerosis under conditions of renal failure due to vascular calcification, elevated serum phosphorous levels, and hyperparathyroidism. Even so, DCB is a superior treatment to percutaneous transluminal angioplasty (PTA) in dialysis patients with femoropopliteal disease, as demonstrated by Hsin-Hua Chou et al. [26].

In addition, restenosis and TASC D lesions also markedly affected primary patency, but the differences were not statistically significant in this study. It is worth mentioning that recent research has found that clinical outcomes after DCB angioplasty in TASC D lesions were inferior to what had been reported and much more unsatisfactory than those in TASC A to C lesions [27]. This result should be considered in clinical treatment, and therefore, long-lesion studies including TASC D lesions are of clinical value. Furthermore, there were also some other potential predictors of primary patency, such as female sex, obesity, lesion length and severe calcification, which were found in other studies but they require confirmation by additional clinical data [28, 29].

Since a meta-analysis reported an increased risk of death following DCBs use in femoropopliteal artery disease [10], the safety of DCBs has remained a hot topic. Other studies, however, have come to a different conclusion. Gray WA et al. found no significant difference in all-cause mortality between DCB and PTA over 3 years, and Schneider PA et al. obtained the same results through 5 years, comparing patients with similar characteristics [11, 30]. In this study, the rate of all-cause death up to 1 year was 2.8% (n = 3 of 109), and none of the deaths were attributable to the DCBs. Our results suggest that DCB applications are safe in long femoropopliteal artery lesions at 1 year, similar to those of other safety studies [11, 30, 31]. Longer-term safety results need to be confirmed by further follow-up.

## Study limitations

This study is single-arm and not randomized, so we cannot compare the effectiveness of DCBs with other treatments on long femoropopliteal lesions, and selection bias is inevitable. The sample size was not large since not all patients with long lesions were treated with DCBs and many were treated with plain stents or DESs, but we increased the sample size through a multicentre study. The patients included were all Asian, and the applicability of the results to different racial groups requires further study. In addition, since the patients are from different cities and usually live far away from the research centres, longer-term follow-up is difficult.

## Conclusion

Our multicentre study demonstrated that Orchid^®^ DCBs were safe and effective at the 1-year follow-up when used in long femoropopliteal lesions in a real-world setting, and the primary patency rate at 12 months by Kaplan–Meier estimation was 82.1%. Renal failure and CLI were baseline predictors affecting the primary patency rate.

## Data Availability

The datasets used and/or analysed during the current study are available from the corresponding author on reasonable request.

## Abbreviations

ABI: Ankle brachial index
CLI: Critical limb ischemia
CTA: Computed tomography angiography
CTO: Chronic total occlusion
DCB: Drug-coated balloon
PTA: percutaneous transluminal angioplasty
TASC: TransAtlantic InterSociety Consensus II
TLR: Target lesion revascularization

## References

[1] Werk M, Albrecht T, Meyer DR, Ahmed MN, Behne A, Dietz U (2012). Paclitaxel-coated balloons reduce restenosis after femoro-popliteal angioplasty: evidence from the randomized PACIFIER trial. Circ Cardiovasc Interv..

[2] Jaff MR, Rosenfield K, Scheinert D, Rocha-Singh K, Benenati J, Nehler M (2015). Drug-coated balloons to improve femoropopliteal artery patency: rationale and design of the LEVANT 2 trial. Am Heart J..

[3] Tepe G, Schnorr B, Albrecht T, Brechtel K, Claussen CD, Scheller B (2015). Angioplasty of femoral-popliteal arteries with drug-coated balloons: 5-year follow-up of the THUNDER trial. JACC Cardiovasc Interv..

[4] Rosenfield K, Jaff MR, White CJ, Rocha-Singh K, Mena-Hurtado C, Metzger DC (2015). Trial of a paclitaxel-coated balloon for femoropopliteal artery disease. N Engl J Med..

[5] Schroeder H, Werner M, Meyer DR, Reimer P, Krüger K, Jaff MR (2017). Low-dose paclitaxel-coated versus uncoated percutaneous transluminal balloon angioplasty for femoropopliteal peripheral artery disease: one-year results of the ILLUMENATE European randomized clinical trial (randomized trial of a novel paclitaxel-coated percutaneous angioplasty Balloon). Circulation..

[6] Schneider PA, Laird JR, Tepe G, Brodmann M, Zeller T, Scheinert D (2018). Treatment effect of drug-coated balloons is durable to 3 years in the femoropopliteal arteries: long-term results of the IN.PACT SFA randomized trial. Circ Cardiovasc Interv.

[7] Gardiner GA, Bonn J, Sullivan KL (2001). Quantification of elastic recoil after balloon angioplasty in the iliac arteries. J Vasc Interv Radiol..

[8] Katsanos K, Spiliopoulos S, Paraskevopoulos I, Diamantopoulos A, Karnabatidis D (2016). Systematic review and meta-analysis of randomized controlled trials of paclitaxel-coated balloon angioplasty in the femoropopliteal arteries: role of paclitaxel dose and bioavailability. J Endovasc Ther..

[9] Mathlouthi A, Yei KS, Naazie I, Bertges DJ, Malas MB. Increased mortality with paclitaxel-eluting stents is driven by lesion length. J Vasc Surg. 2020. 10.1016/j.jvs.2020.05.061 32615286

[10] Katsanos K, Spiliopoulos S, Kitrou P, Krokidis M, Karnabatidis D (2018). Risk of death following application of paclitaxel-coated balloons and stents in the femoropopliteal artery of the leg: a systematic review and meta-analysis of randomized controlled trials. J Am Heart Assoc..

[11] Schneider PA, Laird JR, Doros G, Gao Q, Ansel G, Brodmann M (2019). Mortality not correlated with paclitaxel exposure: an independent patient-level meta-analysis of a drug-coated balloon. J Am Coll Cardiol..

[12] Radke PW, Joner M, Joost A, Byrne RA, Hartwig S, Bayer G (2011). Vascular effects of paclitaxel following drug-eluting balloon angioplasty in a porcine coronary model: the importance of excipients. EuroIntervention.

[13] Banerjee S, Khalili H (2018). Drug-coated balloon for long femoropopliteal lesions. Circ Cardiovasc Interv.

[14] Micari A, Vadalà G, Castriota F, Liso A, Grattoni C, Russo P (2016). 1-Year results of paclitaxel-coated balloons for long femoropopliteal artery disease: evidence from the SFA-long study. JACC Cardiovasc Interv..

[15] Micari A, Nerla R, Vadalà G, Castriota F, Grattoni C, Liso A (2017). 2-Year results of paclitaxel-coated balloons for long femoropopliteal artery disease: evidence from the SFA-long study. JACC Cardiovasc Interv..

[16] Scheinert D, Micari A, Brodmann M, Tepe G, Peeters P, Jaff MR (2018). Drug-coated balloon treatment for femoropopliteal artery disease. Circ Cardiovasc Interv.

[17] Torsello G, Stavroulakis K, Brodmann M, Micari A, Tepe G, Veroux P, et al. Three-year sustained clinical efficacy of drug-coated balloon angioplasty in a real-world femoropopliteal cohort. J Endovasc Ther. 2020:1526602820931477. 10.1177/1526602820931477 PMC7545651 32583749

[18] Li X, Zhou M, Ding Y, Wang Y, Cai L, Shi Z (2020). A systematic review and meta-analysis of the efficacy of debulking devices for in-stent restenosis of the femoropopliteal artery. J Vasc Surg..

[19] Kelsch B, Scheller B, Biedermann M, Clever YP, Schaffner S, Mahnkopf D (2011). Dose response to paclitaxel-coated balloon catheters in the porcine coronary overstretch and stent implantation model. Invest Radiol..

[20] Okuno S, Iida O, Shiraki T, Fujita M, Masuda M, Okamoto S (2016). Impact of calcification on clinical outcomes after endovascular therapy for superficial femoral artery disease: assessment using the peripheral artery calcification scoring system. J Endovasc Ther..

[21] Gerhard-Herman MD, Gornik HL, Barrett C, Barshes NR, Corriere MA, Drachman DE (2017). 2016 AHA/ACC guideline on the management of patients with lower extremity peripheral artery disease: a report of the American College of Cardiology/American Heart Association Task Force on Clinical Practice Guidelines. Circulation..

[22] Mohapatra A, Saadeddin Z, Bertges DJ, Madigan MC, Al-Khoury GE, Makaroun MS (2020). Nationwide trends in drug-coated balloon and drug-eluting stent utilization in the femoropopliteal arteries. J Vasc Surg..

[23] Newman AB, Naydeck BL, Whittle J, Sutton-Tyrrell K, Edmundowicz D, Kuller LH (2002). Racial differences in coronary artery calcification in older adults. Arterioscler Thromb Vasc Biol..

[24] Jia X, Zhang J, Zhuang B, Fu W, Wu D, Wang F (2016). Acotec drug-coated balloon catheter: randomized, multicenter, controlled clinical study in femoropopliteal arteries: evidence from the AcoArt I Trial. JACC Cardiovasc Interv..

[25] Xu Y, Jia X, Zhang J, Zhuang B, Fu W, Wu D (2018). Drug-coated balloon angioplasty compared with uncoated balloons in the treatment of 200 Chinese patients with severe femoropopliteal lesions: 24-month results of AcoArt I. JACC Cardiovasc Interv..

[26] Chou HH, Huang HL, Hsieh CA, Jang SJ, Tzeng IS, Ko YL (2018). Drug-Coated Balloon versus conventional balloon angioplasty in dialysis patients with symptomatic femoropopliteal disease—a matched comparison. Circ J..

[27] AbuRahma AF, AbuRahma ZT, Scott G, Adams E, Beasley M, Davis M (2019). Clinical outcome of drug-coated balloon angioplasty in patients with femoropopliteal disease: a real-world single-center experience. J Vasc Surg..

[28] Schmidt A, Piorkowski M, Görner H, Steiner S, Bausback Y, Scheinert S (2016). Drug-coated balloons for complex femoropopliteal lesions: 2-year results of a real-world registry. JACC Cardiovasc Interv..

[29] Hsieh CA, Chou SH, Chen IC, Jang SJ, Chou HH, Ko YL (2019). Feasibility and mid-term outcomes of drug-coated balloon angioplasty between intermittent claudication and critical limb ischemia in patients with femoropopliteal disease. Acta Cardiol Sin..

[30] Gray WA, Jaff MR, Parikh SA, Ansel GM, Brodmann M, Krishnan P (2019). Mortality assessment of paclitaxel-coated balloons: patient-level meta-analysis of the illumenate clinical program at 3 years. Circulation..

[31] Ouriel K, Adelman MA, Rosenfield K, Scheinert D, Brodmann M, Peña C (2019). Safety of paclitaxel-coated balloon angioplasty for femoropopliteal peripheral artery disease. JACC Cardiovasc Interv..

